# Mucormycosis in Indian COVID-19 Patients: Insight into Its Patho-Genesis, Clinical Manifestation, and Management Strategies

**DOI:** 10.3390/antibiotics10091079

**Published:** 2021-09-06

**Authors:** Ram Kumar Sahu, Mounir M. Salem-Bekhit, Bedanta Bhattacharjee, Yosif Almoshari, Abu Md Ashif Ikbal, Meshal Alshamrani, Alakesh Bharali, Ahmad Salawi, Retno Widyowati, Abdulrahman Alshammari, Ibrahim Elbagory

**Affiliations:** 1 Department of Pharmaceutical Science, Faculty of Pharmacy, Universitas Airlangga, Surabaya 60115, Indonesia; ramkumar.sahu@aus.ac.in (R.K.S.); rr-retno-w@ff.unair.ac.id (R.W.); 2 Department of Pharmaceutical Science, Assam University (A Central University), Silchar 788011, India; 3 Department of Pharmaceutics, College of Pharmacy, King Saud University, Riyadh 11451, Saudi Arabia; 4 Department of Microbiology and Immunology, Faculty of Pharmacy, Al-Azhar University, Cairo 11884, Egypt; 5 Department of Pharmaceutical Sciences, Faculty of Science and Engineering, Dibrugarh University, Dibrugarh 786004, India; bedanta1994@gmail.com; 6 Department of Pharmaceutics, College of Pharmacy, Jazan University, Jazan 45142, Saudi Arabia; Yalmoshari@jazanu.edu.sa (Y.A.); malshamrani@jazanu.edu.sa (M.A.); asalawi@jazanu.edu.sa (A.S.); 7 Department of Pharmacy, Tripura University (A Central University), Suryamaninagar 799022, India; 8 Department of Pharmaceutics, Girijananda Chowdhury Institute of Pharmaceutical Sciences, Azara, Hatkhowapara, Guwahati 781017, India; bharalialakesh99@gmail.com; 9 Department of Pharmacology and Toxicology, College of Pharmacy, King Saud University, Riyadh 11451, Saudi Arabia; abdalshammari@ksu.edu.sa; 10 College of Pharmacy, Northern Border University, Arar 1321, Saudi Arabia; Ibrahim.Elbagory@nbu.edu.sa

**Keywords:** COVID-19, mucormycosis, nanoparticles, pathogenesis, amphotericin-B

## Abstract

Mucormycosis in patients who have COVID-19 or who are otherwise immunocompromised has become a global problem, causing significant morbidity and mortality. Infection is debilitating and fatal, leading to loss of organs and emotional trauma. Radiographic manifestations are not specific, but diagnosis can be made through microscopic examination of materials collected from necrotic lesions. Treatment requires multidisciplinary expertise, as the fungus enters through the eyes and nose and may even reach the brain. Use of the many antifungal drugs available is limited by considerations of resistance and toxicity, but nanoparticles can overcome such limitations by reducing toxicity and increasing bioavailability. The lipid formulation of amphotericin-B (liposomal Am-B) is the first-line treatment for mucormycosis in COVID-19 patients, but its high cost and low availability have prompted a shift toward surgery, so that surgical debridement to remove all necrotic lesions remains the hallmark of effective treatment of mucormycosis in COVID-19. This review highlights the pathogenesis, clinical manifestation, and management of mucormycosis in patients who have COVID-19.

## 1. Introduction

Mucormycosis, previously known as zygomycosis, is a lethal fungus in which molds called mucormycetes can cause fungal infection [1,2,3]. Mucormycosis causes angioinvasive infection among immunocompromised patients, with a mortality rate of 60% [4]. Mucormycosis is the third most prevalent fungal infection in hematology patients, accounting for 8.3–13% of all fungal infections [5,6]. Mucorales fungi access the human body mostly by inhalation, percutaneous contact, or ingestion [7]. Mucormycosis generally occurs in patients who are immunocompromised by leukemia, lymphoma, neutropenia, diabetes, burn, trauma, childhood malnutrition, and the like [8,9]. Diabetes mellitus is the key vulnerability factor related to mucormycosis in India [10], which leads the world in mucormycosis as well as diabetes [11,12]. Mucormycosis is notoriously difficult to diagnose, with Ingram et al. having found that only 9% of cases were identified in antemortem diagnosis [13].

SARS-CoV-2 (severe acute respiratory syndrome coronavirus 2), the virus behind the COVID-19 outbreak, is linked to a variety of bacterial and fungal infections [14]. In India and some other countries, mucormycosis has co-occurred extensively with COVID-19 and is considered to be an epidemic by the Indian government based on reports of the infection mainly affecting hospitalized COVID-19 patients, leading to prolonged morbidity and death. Researchers have found that it chiefly affects immunocompromised patients admitted to the hospital when fungal spores enter a COVID-19–infected person through an airborne vector, affecting the sinuses and lungs, though rarely in persons who have strong immunity. Patients who have been treated for COVID-19 by using steroids and other drugs to cure inflammation are the most vulnerable to mucormycosis [14]. The erroneous administration of corticosteroids (i.e., Prednisone, Hydrocortisone, or Dexamethasone) is a contributing factor to mucormycosis infection in COVID-19 patients. Even though steroids are effective in treating respiratory illnesses including chronic obstructive pulmonary disease and asthma, and other illnesses such as rheumatoid arthritis, the long-term or excessive use of steroids suppresses the body’s immunological system, making the person more susceptible to diseases such as mucormycosis infection [15].

**Figure 1:**
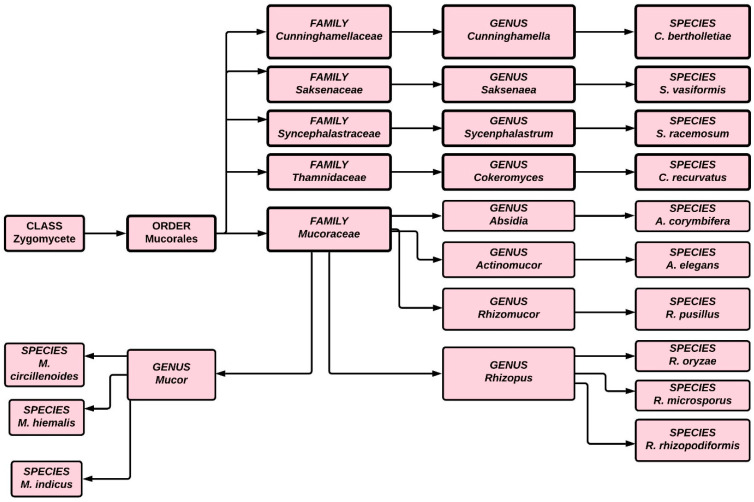
Classification of fungi in the zygomycete order.

The first case of black fungus was reported during the first wave of the COVID-19 pandemic in India, a couple of weeks after the patient’s discharge from a hospital. During the second wave of COVID-19, infections were reported even while patients were undergoing hospital treatment. Although mucormycosis can be treated by antifungal medication, ultimately surgery is required. Traditionally treatment has involved intravenous infusion of regular saline followed by an infusion of amphotericin, but a lack of clinical trial data has hindered researchers and scientists from choosing specific antifungal agents for treating mucormycosis. Because of the high mortality associated with this infection, effective treatment requires early detection and depends on recovery from predisposing factors. The condition can also be improved through surgical debridement and administration of medication [16,17,18]. In India, 28,252 occurrences of mucormycosis, or black fungus, have been documented in 28 states and union territories, with Maharashtra and Gujarat accounting for the overwhelming majority [19]. Figure 1 illustrates the classification of fungi in the zygomycete order.

## 2. Manifestation of Mucormycosis

Mucormycosis symptoms vary depending on where the fungus develops in the body [14,20]. Symptoms of rhinocerebral mucormycosis include black sores on the nasal bridge, fever, one-sided face edema, headache, and nasal congestion, whereas the symptoms of cutaneous mucormycosis are swelling around the wound, pain, and excessive redness. By contrast, the symptoms of pulmonary mucormycosis include breathlessness, chest pain, coughing, and fever. Finally, the symptoms of gastrointestinal mucormycosis include stomach pain, stomach bleeding, and nausea and vomiting.

Because disseminated mucormycosis develops in patients who have been admitted to the hospital for other diseases, determining which symptoms are caused by mucormycosis can be difficult. Eventually such patients may develop mental status changes that may lead to coma. Because some of the symptoms of mucormycosis and COVID-19 are similar, physicians may have difficulty determining whether an individual is infected with a fungus or with COVID-19. Furthermore, certain patients may have COVID-19 along with a fungal infection.

## 3. Epidemiology

According to recent data, the number of reported cases of mucormycosis has increased significantly [8]. For example, the incidence of mucormycosis has increased dramatically in major transplant facilities, with the number of patients more than doubling in 15 years [21,22]. Among autopsied individuals who had leukemia, the incidence of mucormycosis reached 8% in high-risk individuals, with diabetes mellitus reported in 54–76% of cases and diabetic ketoacidosis in 8–22%. In north India, individuals who had diabetes mellitus exhibited a 0.16–1.72% occurrence of mucormycosis [23,24]. Mucormycosis revealed diabetes in 24% of patients in south India, 40% in western India, and 43% in north India, reflecting a lack of routine health checkups among Indian people [25]. In India, 1–9% of mucormycosis patients have a hematological malignancy, compared with 38–62% in the United States and Europe [10,26]. The frequency of confirmed mucormycosis was 1.4% among 781 acute leukemia patients studied in north India [27]. In a study of acute myeloid leukemia patients in south India, the prevalence of verified mucormycosis cases was 0.9% [28]. In India, solid-organ transplantation is a risk factor in 2.6–11% of mucormycosis cases, compared with 7–14% globally [27]. Furthermore, in India, mucormycosis occurs in from 0.05% to 2.7% of renal transplant patients, versus 0.04–0.05% globally [29,30]. According to many investigations, mucormycosis is found in 0.56–1.52% of kidney transplant recipients in south India [31,32]. Steroid treatment, chronic obstructive pulmonary disease, chronic renal illness, and pulmonary tuberculosis are all risk factors for mucormycosis in India, where chronic kidney disease has emerged as a new risk factor for mucormycosis [33,34]. According to Indian studies, 9–32% of mucormycosis patients have chronic kidney disease [35]. Likewise, chronic obstructive pulmonary disease and pulmonary tuberculosis have been reported in 7–46% of individuals who had mucormycosis [35].

Low birth weight babies, chronic alcoholism, liver diseases, renal failure, intravenous drug use, malnutrition, and acquired immunodeficiency syndrome are all factors associated with mucormycosis [12]. Mucormycosis is exceedingly uncommon in HIV-positive individuals. Only two individuals developed mucormycosis in a retrospective analysis of 1630 autopsy of AIDS patients who died between 1984–2002 [36]. The most prevalent comorbidities were the usage of corticosteroid (25%), neutropenia (29.7%), and intravenous drug use (IVDU 50%). Individuals with a record of intravenous drug use who experienced mucormycosis are more likely to present with localized cerebral inflammation [37]. In Indian research published in 2019, chronic renal illness (8.9%) and post-pulmonary TB (6.9%) were identified as rising risk factors [31].

## 4. Pathogenesis Mechanism

### 4.1. Phagocytes

**Figure 2:**
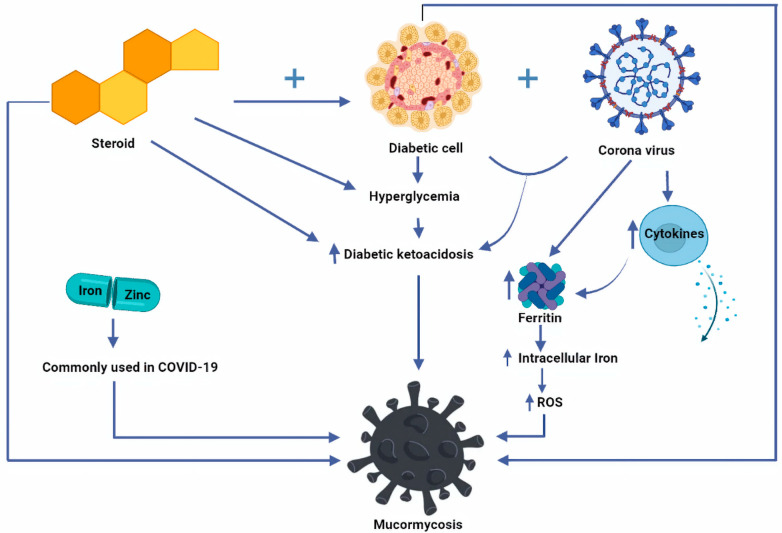
Pictorial representation of etiopathogenesis of mucormycosis in COVID-19 patients.

Mononuclear cells, macrophages, and neutrophils make up the second and most significant line of defense against intruding fungus. This immune system barricade is essentially twofold: Tissue macrophages aid in spore phagocytosis, whereas spores that escape and develop into hyphae cause neutrophil chemotaxis, which has an oxidative cytotoxic effect [38,39]. These cells directly kill as well as phagocytose spores and hyphae by producing and releasing perforin, antimicrobial enzymes, reactive oxygen metabolites, and cationic peptides [40]. They also release pro-inflammatory cytokines such as tumor necrosis factor (TNF)-α, interleukin-1 beta (IL-1b), and interferon-gamma (IFN-γ) which activate and attract other immune cells. Interruption or failure of this initial inflammatory response can cause tissue damage and infection dissemination [41]. Pro-inflammatory cytokines are decreased in COVID-19 patients, allowing the fungal infection to spread more widely. Fungi adhere to phagocyte particular pattern receptors via diverse pathogen-associated chemical patterns, causing activation and propagation of intracellular signals [42]. Toll-like receptors, particularly Toll-like receptor 2, are essential in the early stages of the pro-inflammatory response [43]. Numerous host conditions might impair phagocyte functioning, allowing Mucorales to invade more easily. Studies have indicated that corticosteroid treatment makes mouse pulmonary alveolar macrophages incapable of preventing sporangiospore germination [44]. In another work, simulated ketoacidosis circumstances inhibited phagocyte cytotoxicity and increased *R. oryzae* development, a result that was entirely recovered after acidosis was corrected [45]. In neutropenic patients who have mucormycosis, the Third European Conference on Leukemia Infections recommends use of granulocyte–macrophage colony-stimulating and granulocyte colony-stimulating factors in neutropenic patients only [46]. Figure 2 illustrates the etipathogenesis of mucormycosis.

### 4.2. Platelets

**Figure 3:**
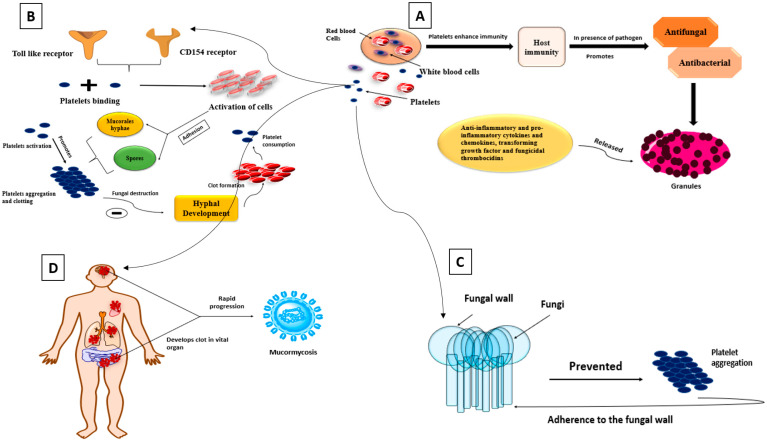
Schematic diagrams of platelets roles; (**A**) Platelets enhance host immunity, and after invading pathogen/foreign matter it promotes antifungal and antibacterial properties. Thus, various cytokines and chemokines released in granules; (**B**) The two membrane bound molecules such as TLR and CD154, it allows platelets binding and activates different cells. Thus, adhesion of mucorales hyphae and spores takes place, which causes platelets activation and leads to aggregation and clotting. Furthermore, inhibits hyphal development in presence of fungal destruction, which leads to clot formation and platelets consumption; (**C**) Prevention of fungi takes place due to the presence of platelet aggregation and therefore, it adheres to fungal wall; (**D**) COVID-19 patient develops clot in their vital organ and therefore progression of mucormycosis becomes rapid.

Platelets play an essential function in enhancing host immunity [46] and exhibit antifungal and antibacterial functions after exposure to an invading pathogen: Anti-inflammatory and pro-inflammatory cytokines and chemokines, including transforming growth factor and fungicidal thrombocidins, are released in granules [47]; platelet Toll-like receptors and CD154 are membrane-bound molecules that allow platelet binding and activation of different cells [48,49]; and adhesion to Mucorales hyphae and spores causes platelet activation, promotes platelet aggregation and clotting, as well as fungal destruction, by inhibiting hyphal development, supporting the development of clots and causing platelet consumption (Figure 3) [50,51]. Fungi may also be prevented from spreading hematogenously by platelet aggregation and adherence to the fungal wall [49]. Necrotic areas were discovered in tissues with no obvious fungal development, implying that thrombotic ischemia occurred due to systematic platelet activation [50]. Patients with COVID-19 who have the severe form of the disease frequently develop clots in vital organs, which can lead to further complications. As a result, the progression of mucormycosis is quite rapid. 

### 4.3. Natural Killer Cells

**Figure 4:**
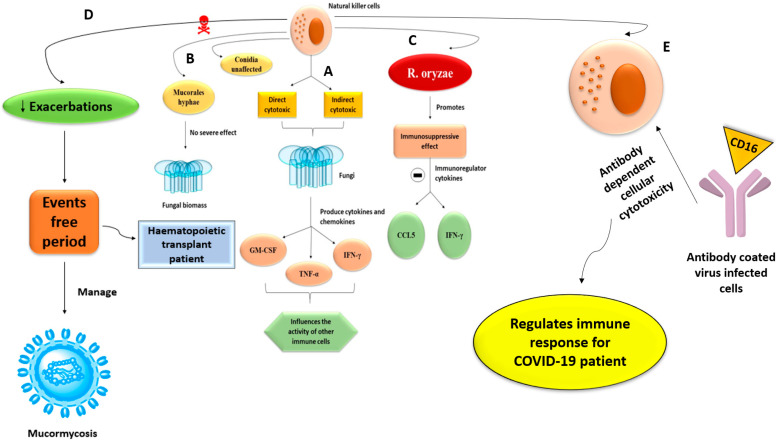
Schematic diagrams of natural killer cells roles; (**A**) Natural killer cells have direct and indirect cytotoxic effects on fungi and produce cytokines and chemokines (includes GM-CSF, TNF-α, IFN-γ. Thus, influences the activity of other immune cells; (**B**) Damage of mucorales hyphae takes place in presence of natural killer cells, but no effects shown in conidia. Therefore, it leads to no fungal infection; (**C**) *R. oryzae* promotes immunosuppressive effects and thus, it inhibits the release of immune regulatory chemokines such as CCL5 and IFN-γ; (**D**) Human natural killer cells are studied for their ability to minimize exacerbations and enhance event-free periods in hematopoietic transplant patients, and their therapeutic effect may also be helpful in managing and providing therapy for invasive mucormycosis; (**E**) Natural killer cell counts can be severe to the IgG immunity for COVID-19. The involvement of CD16 on natural killer cells by antibody-coated virus-infected cells results in antibody-dependent cellular cytotoxicity, thus regulates immune response for COVID-19 patient.

Natural killer cells are a type of innate immune cell that have both direct and indirect cytotoxic effects on the fungi. They also produce cytokines and chemokines such as GM-CSF, TNF-α, and IFN-γ, which influence the activity of other immune cells. Mucorales hyphae can be damaged by natural killer cells, but conidia are unaffected. Moreover, the damage done is inversely proportional to the amount of fungal biomass and has nothing to do with fungal infection. Conversely, in vitro investigations have shown *R. oryzae* to have an immunosuppressive effect, inhibiting the release of immunoregulatory chemokines RANTES (CCL5) and IFN-γ from natural killer cells [52]. Human natural killer cells are studied for their ability to minimize exacerbations and enhance event-free periods in hematopoietic transplant patients, and their therapeutic effect may also be helpful in managing and providing therapy for invasive mucormycosis (Figure 4) [53,54]. COVID-19 IgG immunity can be severely impaired by high natural killer cell numbers. Antibody-coated virus-infected cells interact with CD16 on natural killer cells, resulting in antibody-dependent cellular cytotoxicity [55]. The innate immune system controls immunological response and acts as a first line of defense against COVID-19.

### 4.4. Iron Uptake

**Figure 5:**
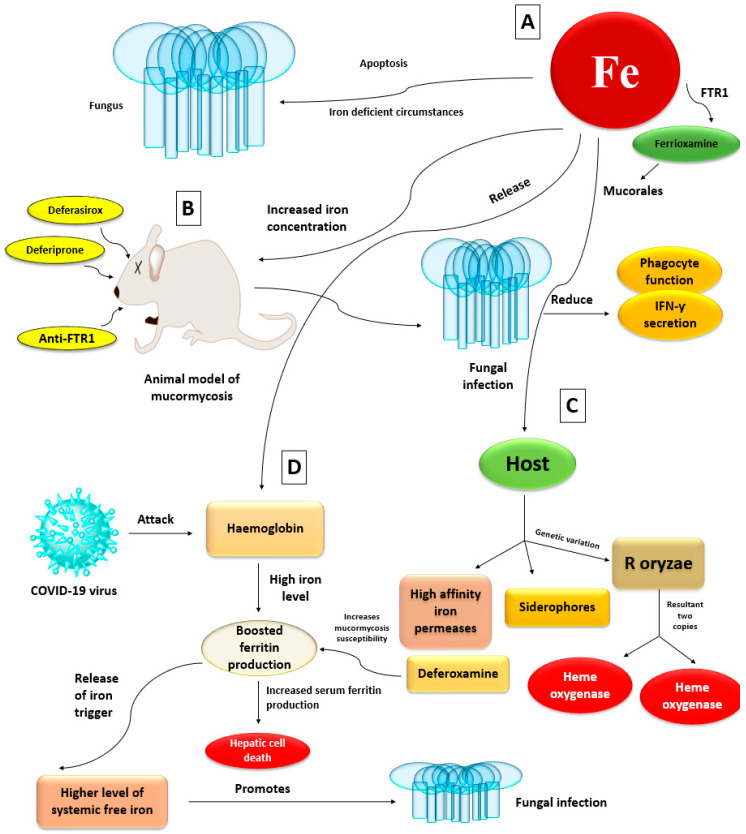
Schematic diagrams of Iron uptakes roles; (**A**) Higher level of Iron (Fe) leads to fungal growth, whereas fungal undergoes apoptosis in iron deficient circumstances; (**B**) Increase level of iron in animal model cause fungal infection, due to the reduction of phagocyte function and IFN-γ; (**C**) In the host body the Mucorales obtain iron via three mechanisms: High affinity iron permeases, siderophores and *R. oryzae*. The *R. oryzae* promotes the existence of two copies of hemeoxygenases; (**D**) COVID-19 virus attack haemoglobin and in the presence of higher level of iron it boosted ferritin production, which leads to the increase serum ferritin production and causes hepatic cell death. Therefore, release of iron trigger and leads to higher level of systemic free iron and cause fungal infection. Thus, various drugs show beneficial effects in the management of Mucorales such as deferasirox, deferiprone ferrioxamine and anti-FTR1.

The fungus undergoes apoptosis in iron-deficient circumstances, supporting iron’s necessity for fungal cell growth [54]. In animal models of mucormycosis, increased iron concentrations also aid fungal development by reducing phagocyte function and lowering IFN-γ secretion [56]. Mucorales obtain iron from their hosts via two major mechanisms: high-affinity iron permeases or siderophores [57]. In addition, genetic investigation of *R. oryzae* revealed the existence of two copies of heme oxygenases, implying a third mechanism of iron acquisition from hemoglobin [58]. The COVID-19 virus may target haemoglobin, causing iron to be released from porphyrins and into the circulation, resulting in iron overload. Ferritin production is increased to compensate for the high iron level. Increased serum ferritin levels can promote hepatic cell death, causing ferritin to release iron, resulting in greater levels of systemic free iron [59]. As a result, it promotes the spread of fungal infection. Deferoxamine, an iron chelator used in those at risk of iron overload, increases mucormycosis susceptibility [60]. Mucorales use ferrioxamine (the iron-rich form of deferoxamine) as a xenosiderophore to collect iron [61]. Various researchers have demonstrated that iron chelation therapy with deferasirox or deferiprone prevents mucormycosis in mice with diabetic ketoacidosis and promotes longevity, whereas adjunctive deferasirox was both effective and tolerated in an open-label study of eight cases of mucormycosis [62,63]. Deferasirox also countered iron’s inhibitory effect on neutrophil chemotaxis [64]. However, recent clinical research in individuals who had mucormycosis that used supplementary deferasirox medication failed to establish a survival effect [65]. FTR1, a high-affinity iron permease that facilitates iron absorption, is upregulated in an iron-depleted state and silenced in iron-rich situations [66]. It has been proposed that FTR1 facilitates the intracellular transfer of iron from ferrioxamine or heme. An experimental investigation demonstrated that anti-FTR1 antibodies protected mice with diabetic ketoacidosis from infection, highlighting its potential as a therapy target (Figure 5) [67].

### 4.5. Interplay with the Endothelium

Mucorales bind to the surface of the endothelial cells by releasing proteins known as spore coat homologs [68], which are present only in Mucorales and interact with the host endothelium receptor GRP78, causing the fungus to endocytose [41,68]. GRP78, also known as heat shock protein, is found mainly in the endoplasmic reticulum and is presumed to become a specialized host cell receptor [60]. Spore coat homolog protein fungal surface expression and GRP78 endothelium surface expression increase after endothelial cells are confronted with acidosis, high glucose, and high iron levels, as in diabetic ketoacidosis high blood sugar. Thus COVID-19 patients with diabetes comorbidities can increase fungal endocytosis through the endothelium. In one study, GRP78 and spore coat homolog proteins were elevated by acidosis induced by β-hydroxybutyrate (a ketone body) but not affected by higher blood iron levels lactic acidosis. Furthermore, sodium bicarbonate recovered acidosis and protected β-hydroxybutyrate–treated mice from developing mucormycosis, indicating the importance of restoring acidosis as a therapeutic strategy in patients with mucormycosis and diabetic ketoacidosis. Anti-spore coat homolog protein and anti-GRP78 antibodies were used in another investigation to largely prevent *R. oryzae* endothelium invasion [43].

### 4.6. Voriconazole Exposure

Clinically relevant epidemiologic data reveal that voriconazole is an antifungal commonly used for prophylaxis in high-risk patients but is ineffective against Mucorales [69]. Voriconazole caused hypervirulent Rhizopus and Mucor strains in mice, increasing lung fungal loads and shortening lifespans [43]. The hypervirulent phenotype was lost when voriconazole treatment stopped, indicating an epigenetic rather than a genetic shift [70]. Voriconazole is currently recommended as first-line therapy for COVID-19 associated pulmonary aspergillosis (CAPA), which can lead to the fast spread of mucormycosis [71]. A recent investigation using rat models found that pre-exposure of fungal spores to voriconazole produces breakthrough infections by *R. oryzae*, which appear to be less responsive to subsequent antifungal therapy [72]. If voriconazole exposure does indeed select for more virulent strains, then we may be only beginning to understand the processes that contribute to Mucorales pathogenicity.

## 5. Mucormycosis Outbreak

An outbreak occurs when two or more people are infected by the same source or at the same place or time. The sources of outbreaks may be outdoors or may be in a healthcare setting, such as a hospital [73]. Most published works describe outbreaks of cutaneous mucormycosis, which has been tied to contaminated dressings and is less fatal than other forms of mucormycosis, with a medical literature review revealing 16% mortality versus 67% for rhinocerebral, 83% for pulmonary, and 100% for disseminated and gastrointestinal mucormycosis [74]. Hospital bedding has been identified as a vector for spreading *R. delemar* to vulnerable patients. In hospital epidemic investigations, DNA-based approaches to fungal species detection have confirmed epidemiological connections. Hospital bedding must be washed, wrapped, distributed, and stored in ways that minimize their exposure to environmental pollutants [75].

## 6. Effects of Black Fungus on COVID-19 Patients

The outbreak of mucormycosis is yet another unpleasant surprise brought on by the COVID-19 pandemic [76]. It can infect the sinuses and facial bones, infiltrate the brain, and lead to the loss of an eye. If left untreated, mucormycosis kills up to half of patients—and treatment is time-consuming and difficult. The standard treatment approach for severe COVID-19 is a high dose of steroids, antibiotics, and antivirals, which dampen the immune system and make the patient susceptible to infection by bacteria and fungus already present in the body or the environment [77]. Infection with mucormycosis is akin to opening Pandora’s box. Having been infected by SARS-CoV-2, the patient’s body is already ravaged, and infection by a virulent fungus often leads to death.

To save a patient’s life, doctors may surgically remove an organ or tissue from the body, causing emotional anguish for the patient and family members. Moreover, the standard treatment for black fungus is Amphotericin B, which is exorbitantly expensive, to the point that insolvency may arise from treatment [78].

## 7. Diabetes Patients Are Predisposed to Mucormycosis

**Figure 6:**
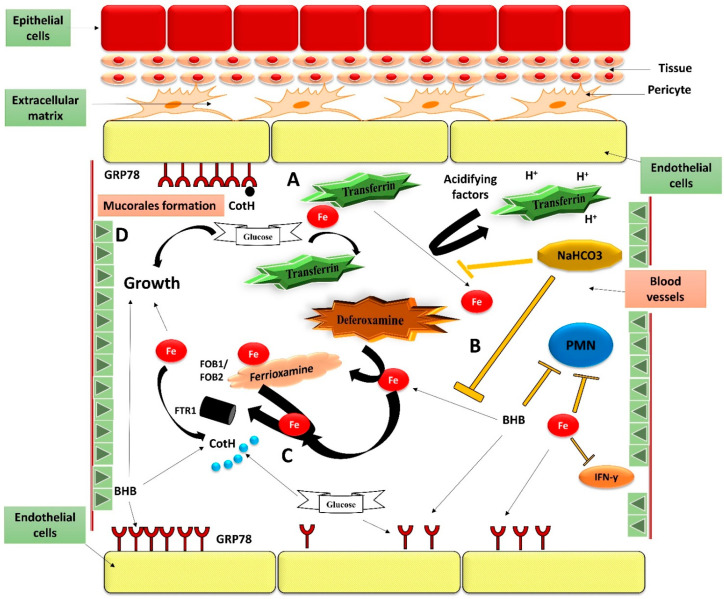
Interaction between Mucorales and endothelial cells during hematogenous proliferation, as well as the influence of host attributes on these relationships and the immune response. (**A**)—Through glycosylation and protonation, hyperglycemia and ketoacidosis trigger the production of Fe from transferrin.; (**B**)—Immunological response to disease is harmed by BHB and free Fe, but at the other end, NaHCO3 counteracts this damaging activity by decreasing transferrin iron release and mitigating acidity.; (**C**)—GRP78 expression on vascular endothelium significantly increased in response to the stressors caused by free Fe, hyperglycemia and ketone substances.; (**D**)—Free Fe, glucose and BHB increase the production of CotH in fungal cells, resulting in endothelium invasion and increased fungal growth.

Diabetic ketoacidosis and deferoxamine-treated individuals are prone to mucormycosis in a unique way. Diabetic individuals have a high glucose level in their blood [79]. Excessive glycosylation of proteins like ferritin and transferrin might cause them to lose their iron affinity, which further induces hyperglycemia [80]. Furthermore, when there is an acidic state in the blood vessels owing to the build-up of ketone bodies (e.g., β-hydroxybutyrate), transferrin’s potential to chelate iron is severely impaired [81]. β-hydroxybutyrate, glucose, and iron promote fungal development (Figure 6) [56,82]. They also increase the expression of spore coating protein (CotH) and glucose regulator protein78 (GRP78), which leads to increased fungal invasion and consequent endothelial damage in-vitro [70,82]. β-hydroxybutyrate related acidosis appears to have a direct influence on CotH and GRP78 expression (an effect not observed with lactic acid) as well as an indirect effect on transferrin’s potential to chelate iron, as iron chelation coupled with sodium bicarbonate pH reversal substantially protects endothelial cells against Rhizopus-mediated invasion and damage [82]. Notably, when mice administered with β-hydroxybutyrate or diabetic ketoacidosis mice have shown lower blood pH, higher accessible serum iron, higher GRP78 expression in target organs (e.g., sinuses and lungs), they are more vulnerable to mucormycosis [56,82]. It is also important to highlight that optimum levels of β-hydroxybutyrate, iron, and glucose facilitate fungal growth while suppressing the host immune response via phagocyte-mediated destruction, IL-γ generation, and T-lymphocyte activation (Figure 6) [56,82,83,84,85]. Thus, the particular propensity of diabetic ketoacidosis patients to mucormycosis is explained by the idiosyncratic interactions of CotH and GRP78 proteins, as well as their increased expression under hyperglycemia and ketoacidosis conditions [58]. Therapeutic intervention with either anti-CotH or anti-GRP78 antibodies protects neutropenic and diabetic ketoacidosis mice against mucormycosis, highlighting the significance of CotH/ GRP78 protein interactions in the pathophysiology of mucormycosis [69,70,85].

## 8. Implement Control and Preventive Measures

Mucormycosis is an aggressively growing infection that follows infection by COVID-19. Its radiographic manifestations are not specific, but diagnosis can be made by microscopic examination of materials collected from necrotic lesions. Treatment requires multidisciplinary expertise, as the fungus enters through the eyes and nose and can even reach the brain [86]. It can be treated or controlled (1) medically, by using antifungal therapies; (2) surgically, by removing all necrotic lesions; or (3) by implementing adjunctive therapies that reverse the risk factors [87].

## 9. Medical Management of Mucormycosis 

### 9.1. Management by Using Antifungal Drug Therapies 

The lipid formulation of amphotericin-B (liposomal Am-B) is the first-line treatment for mucormycosis in COVID-19 patients. Injection of liposomal Am-B, with a starting dosage of 5–7.5 mg/kg/day, diluted in 500 mL of 5% dextrose over 4–5 h for 14–21 days, is commonly used in hospitalized adults and children [88]. Patients who are intolerant of or unresponsive to Am-B can be given alternative agents, such as an oral suspension of posaconazole, 400 mg two times a day or 200 mg four times a day. However, posaconazole alone cannot be recommended as a primary treatment in patients who have mucormycosis [89]. With progressive infection, monotherapy using lipid Am-B, increasing liposomal Am-B dosage (7.5–10 mg/kg per day), addition of posaconazole or an echinocandin, and shifting to posaconazole are viable strategies for treatment [90]. The evidence does not support combination therapy for first-line healing of mucormycosis, but if first-line therapy fails, a salvage therapy combining a polyene and an echinocandin can be used [89].

Liposomal Am-B is considered an efficient treatment for mucormycosis. Amphotericin B binds to ergosterol present in the fungal cell membrane, which causes pores and subsequent ion leakage, followed by the death of the fungal cells. Several studies have demonstrated the in vitro and in vivo binding of liposomes (both amphotericin B loaded liposomes and empty liposomes) to pathogenic fungal cell walls with gold and fluorescent-labeled liposomes [91,92,93]. As long as a liposome does not have Am-B, it binds to the fungus’s cell wall, but both the fungus cell and liposome stay intact. Liposomes containing amphotericin B, however, are capable of killing fungal cells, which implies that the binding causes the liposome to rupture and release amphotericin B. It then binds to ergosterol in the fungus cell membrane, exerting its fungicidal effect [91]. However, the exact mechanism of how amphotericin B crosses the fungal membrane from the liposome is still unclear. As ergosterol is the primary lipid component of the liposome, amphotericin B likely has a higher affinity for it than cholesterol [94]. As a bonus, liposomal Am-B formulations penetrate biofilms far better than conventional Am-B [95,96]. Despite the excellent pharmacokinetic and pharmacodynamic activity of liposomal Am-B, doctors are shifting towards surgery to save the life of patients because of the poor pharmacoeconomic status of liposomal Am-B. High cost and low availability in the market can be the only limitation of liposomal Am-B [90]. Liposomal Am-B will be called the standard gold drug for treating mucormycosis in COVID-19 patients if such limitations are resolved.

Isavuconazole is the first triazole drug approved by the U.S. Food and Drug Administration (FDA) to treat mucormycosis. It inhibits the CP450-dependent 14-lanosterol demethylase in the fungal cell membrane. As a result, cytotoxic sterols accumulate and reduce ergosterol production, which is essential for fungal cell membrane development. It inhibits fungal growth and replication, eventually leading to cell death [97].

The major drawback of this drug is its resistance like other azoles. Resistance occurs after repeated exposure to the drug. Azole resistance mechanisms include overexpression of ABC transporters (ATP binding cassettes), mutation of the gene encoding the target enzyme (ERG11), ERG3 gene mutation which impairs azole-mediated cell membrane disruption [98]. Therefore, it is a reasonable treatment option for mucormycosis patients with other refractory disorders and posaconazole intolerance [99].

Azoles primarily target ergosterol, which ensures membrane fluidity, permeability, and the proper functioning of membrane proteins [100]. It primarily works by inhibiting ergosterol biosynthesis via the fungal cell membrane’s CP450-dependent 14α-lanosterol demethylase, which is responsible for converting lanosterol to ergosterol. Consequently, the integrity of the fungal cell membrane gets altered, affecting its morphology and growth, eventually leading to cell death [101].

### 9.2. Surgical Management

The high cost and low availability of liposomal Am-B have prompted doctors to conduct surgeries to save the life of patients [90]. Surgical debridement to remove all necrotic lesions remains the hallmark of effective treatment of mucormycosis in COVID-19 patients. Extensive surgery should be conducted as early as possible, with an MRI or CT scan used preoperatively to determine the extent of the tissues in question and the involvement of tissue margins. Repeated surgical removal of necrotic lesions has shown improved outcomes. After successful treatment, the patient may undergo plastic surgery [90]. Surgical recommendations differ by site and by the severity of the condition. Table 1 shows the mode of action, advantages and disadvantages of various medications, as well as surgical management for mucormycosis infection healing.

## 10. Adjunctive Therapies

Along with antifungal agents and surgeries, adjunctive therapies involving reversal of immunosuppression, correction of metabolic deficits, and strategies for immune augmentation are beneficial for controlling mucormycosis [120]. Popular adjunctive therapies include hyperbaric oxygen, immunomodulation strategies, and iron chelation [89]. Hyperbaric oxygen, which is an effective treatment for diabetic patients who have rhinocerebral or severe cutaneous mucormycosis [121], increases the partial pressure of oxygen and improves neutrophilic function. High oxygen concentrations also improve wound healing by releasing enhanced tissue growth factors [122]. However, such therapy has not been subjected to detailed study and so is not regularly recommended. Accordingly, immune augmentation strategies such as granulocyte colony-stimulating factor (G-CSF) and interferon- γ have been implemented as adjunctive therapies to improve host response [123]. Iron chelators such as deferasirox and deferiprone have also been shown to prevent fungal growth and protect diabetic mice from developing mucormycosis. Case reports indicate that iron chelation therapy is a beneficial adjunctive therapy in diabetic patients, whereas a small double-blinded, placebo-controlled, multi-centered study of 20 patients who had a hematologic disorder showed adverse effects when adding deferasirox to liposomal Am-B. Although the study size was limited, the data did not support a role for deferasirox as an adjunctive therapy [124].

## 11. Preventive Measures

The Indian Council of Medical Research (ICMR) has released a set of general guidelines for mucormycosis prevention in COVID-19 patients [125]:

- Good control of sugar level during COVID-19 with or without use of steroids
- Rational use of steroids in the correct dose, with proper timing, and for a suitable duration
- Judicious use of antibiotics/antifungals
- Use of sterile or clean water as humidifiers during oxygen therapy

Additionally, modest preventive actions are indicated for post–COVID-19 recovery patients to avoid mucormycosis:

- Maintaining personal hygiene by thoroughly bathing and scrubbing the body
- Wearing face masks and face shields while visiting dirty or polluted environments
- Wearing concealed shoes, long trousers, long-sleeved shirts, and gloves while handling soil, manure, moss, and the like (especially while gardening)

Because the literature lacks sufficient data on measures for preventing mucormycosis in COVID-19 patients, healthcare professionals follow such general environmental infection control measures to prevent mucormycosis in COVID-19 patients.

Apart from these strategies for managing and preventing mucormycosis in COVID-19 patients, the Ministry of Ayurveda, Unani, Siddha, and Homeopathy (Ayush) in India has suggested different preventive medicines to patients who have recovered from COVID-19 and to those who are on high doses of steroids and are diabetic [126]. The Ministry of Ayush has claimed that ayurvedic formulations can help control black fungus disease among COVID-19 patients, with Unani and homeopathic medicines useful in preventing and treating mucormycosis in COVID-19 patients [127].

## 12. Nanoparticle Formulation of the Drug/Nanoformulation of the Antifungal Drugs

Mucormycosis occurs worldwide, producing severe morbidity and mortality in COVID-19 and other immunocompromised patients. Many efficient antifungal medications are on the market, but their effectiveness is constrained by resistance and considerations of toxicity. Nanoparticles can overcome such limitations by reducing toxicity and increasing bioavailability. Use of nanotechnology for antifungal therapy began in the 1990s with the launch of a lipid formulation of amphotericin B (Am-B) [128]. Apart from the lipid formulation, alternative formulations such as Am-B colloidal dispersion and liposomal Am-B are available [129] and have been found to be safe and effective compared with conventional Am-B. Traditional Am-B is typically prepared as a colloidal suspension for parenteral delivery with sodium deoxycholate, but renal toxicity and severe infusion-related problems hamper its therapeutic efficacy [130,131]. Nanoformulations significantly reduce the toxic effect of the drug but are highly expensive and are available only in parenteral form, limiting their widespread use even apart from the deleterious effect of COVID-19 on personal finances. At 6000–8000 INR, a vial of liposomal Am-B is out of reach of ordinary people, so development of a cost-effective antifungal nanoformulation is an active area of research. Several nanosystems for oral, topical, vaginal, ocular, and pulmonary delivery of Am-B are currently under development and have shown promising in vitro results [132]. Disappointingly, however, only the nano form of Am-B has completed clinical trials. Research into delivery of antifungal medications should address the challenges to clinical translation of nanoparticle-based formulations so that diseases such as mucormycosis and COVID-19 can be combated [133].

## 13. Earlier Diagnose Mucormycosis to Overcome the Adverse Effect

Diagnosing mucormycosis appropriately and undergoing the necessary tests is fundamental to the success of the treatment. Patients with malignant hematological conditions whose amphotericin B-based therapy is delayed beyond the first five days of symptoms have double the 12-week mortality rate [132]. Early diagnosis is therefore crucial to ensuring effective treatment in people with mucormycosis [134,135]. Mucormycosis is currently diagnosed mostly through the culture of the organism from generally sterile body locations and/or histopathologic examination of the affected tissue [89,136]. Medium such as Sabouraud-dextrose agar can be used to isolate mucorales, and the fungal invasion can be investigated using methenamine-silver, eosin, and hematoxylin, calcofluor white stain or periodic acid-Schiff [137]. However, these techniques do not provide enough sensitivity, and end up leading to wrong diagnosis. Computed tomography (CT) can be used to detect pulmonary mucormycosis earlier in cancer patients. In the absence of airway-invasive characteristics, a reversed halo sign can aid in distinguishing the condition from invasive pulmonary aspergillosis in patients with a hematologic malignancy or neutropenia [138]. Radiological indicators of disease are frequently suggestive, rather than diagnostic. Recent development in molecular diagnostic technologies, such as the advent of polymerase chain reaction (PCR)-based tests, along with Matrix Assisted Laser Desorption/Ionization-Time of Flight Mass Spectrometry (MALDI-TOF MS), may assist in the earlier diagnosis of mucormycosis as well as the commencement of treatment [139,140].

## 14. Mucormycosis Diagnosis Limitations in Patients Infected with Microbial Infection

Given the limited treatment choices available, which usually include disfiguring and painful operations, early and accurate diagnosis is, in theory, the most important factor in improving the outcome of mucormycosis. Furthermore, approximately 4 to >90% of suspected mucormycosis cases are not verified until post-mortem investigation [141,142,143]. A combination of variables, the non-specific clinical appearance of mucormycosis, as well as the various limitations of currently available diagnostic techniques, make a definite diagnosis challenging. It is critical to isolate the fungus and identify it to the genus or species level for prognostic, epidemiological, and therapeutic objectives. [142,144,145]. The cultural isolation output ranges from 50–71%, with evidence that it has improved considerably over time [37,141]. Mucorales recovery from clinical microbiology specimens, on the other hand, is difficult. Mucorales hyphae may be difficult to see on wet mounts and require special chitin-binding stains to be seen using a fluorescence microscope, or they may be too few to see. Furthermore, vigorous homogenization or tissue grinding may obliterate the coenocytic hyphae during tissue processing [142,146]. Nonculture techniques, such as detecting biochemical or serological indicators, are currently unavailable to aid in diagnosing invasive mucormycosis. Invasive candidiasis is diagnosed using circulating mannan antigen and (1-3)-β-d-glucan, whereas invasive aspergillosis is diagnosed using galactomannan in bronchoalveolar fluid and serum.

## 15. Future Perspective

Mucormycosis infection is unpleasant, debilitating, and fatal. It leads to loss of organs and emotional trauma in patients, lowering quality of life, and treatment is prohibitively expensive. Amid the COVID-19 outbreak, prevention is of the utmost importance, as is ensuring the availability of an efficient, cost-effective treatment. Despite significant progress in understanding the microbiological aspects of this infection, the COVID-19 outbreak has underscored the need for more awareness, improved diagnostic tests, a focus on diabetes control, and prudent use of corticosteroids, with patients requiring immediate surgery and antifungal treatment. Further research into using modern tools and approaches to prevent and treat this infection is required, along with exploration of novel ways of delivering antifungal drugs that can increase their therapeutic efficacy.

## 16. Conclusions

Fungal infections subsequent to COVID-19 have been observed so extensively in many countries that doctors are beginning to design therapeutic strategies to counteract their effects. Fungal spores are everywhere, but human lungs are generally efficient at clearing them out. However, COVID-19 causes lung damage, diminishing the capacity to naturally eliminate spores in patients who are suffering a weakened immune response as a result of steroid treatment. People who have uncontrolled diabetes are more susceptible to black fungus infection, but the early signs of mucormycosis can be challenging to detect. Unlike some other fungal diseases, it cannot be detected through blood testing. Diagnosis requires a biopsy, examination of the sample, and in some cases a CT scan, all of which require specialized personnel and advanced technology—neither of which can be guaranteed in under resourced areas of India’s healthcare system.

## Figures and Tables

**Table 1:** Demonstrated mode of action, advantages and disadvantages of various medications, as well as surgical management for mucormycosis infection healing.

Treatment Protocol	Mode of Action	Advantages	Disadvantages	Ref
**Therapeutic Intervention**				
Lipid formulation of Amphotericin B (Polyenes derivatives)	The cellular membrane is substantially targeted by Amphotericin B loaded liposomes, which induce fungicidal activity by binding to ergosterol in the fungal cell membrane	➢ Amphotericin B lipid formulations have a higher therapeutic index ➢ Least nephrotoxic, and have better CNS penetration than amphotericin B deoxycholate ➢ Used as first-line treatment for mucormycosis	➢ Hepatotoxicity, infusion-related toxicity ➢ Broad use is limited by the expensive cost and requirement for parenteral administration	[89,102,103]
Posaconazole with or without lipid polyenes (Triazoles derivative)	Inhibits fungal lanosterol 14 α-demethylase enzyme synthesis	➢ Posaconazole oral dosage is simple and convenient ➢ 60–70% success rates were found in a retrospective case study (complete plus partial response) ➢ Posaconazole is an off-label medication for mucormycosis in people who are resistant to amphotericin B	➢ In murine model, the effectiveness of Posaconazole monotherapy was lower than that of polyenes ➢ Posaconazole oral suspension has a reduced absorption rate	[104,105]
Isavuconazole (A new broad-spectrum triazoles derivatives)	Cytochrome P450-dependent lanosterol 14-demethylase enzyme, which is required for the production of ergosterol, a component of the fungal membrane, is inhibited by isavuconazole	➢ High oral absorption; less drug-drug interaction; no need for therapeutic drug monitoring; and linear pharmacokinetics ➢ Isavuconazole has a wide range of fungicidal activity and a low risk of side effects	➢ Hepatotoxicity properties limited the use of Isavuconazole ➢ Isavuconazole has a limited number of clinical studies	[106,107,108,109]
VT-1161	Potent inhibitor of CYP51 and possess in-vitro activity against *Mucorales*, including *Cunninghamella*, *Lichtheimia*, and *Rhizopus oryzae*	➢ It prevented *Rhizopus delemar* infection in immunocompromised mice, and possess modest in-vitro activity against *Mucorales* ➢ VT-1161 has a lower toxicity potential than existing azoles and polyenes, as well as better pharmacokinetics ➢ VT-1161 increased dose-dependent drug plasma levels as well as increased survival rates.	➢ VT-1161 is expensive	[110]
APX001A	The inositol acyltransferase suppressed by APX001A, which limits the development of Glycosylphosphatidylinositol-anchored proteins and producing antifungal effect	➢ Contribute a significant role in the controlled of infectious illness	➢ APX001A has a limited number of clinical studies	[111,112]
Caspofungin plus lipid polyene	Inhibit the enzyme β-1,3-d-glucan synthase	➢ Synergistic in murine disseminated mucormycosis; and favorable toxicity profile	➢ Very limited clinical data of combination therapy	[113,114]
Anidulafungin plus lipid polyene	Inhibit the enzyme β-1,3-d-glucan synthase.	➢ Synergistic with liposomal amphotericin B in murine model of disseminated mucormycosis; and favorable toxicity profile	➢ No clinical data	[115]
Deferasirox plus lipid polyenes	Reducing the available iron load and thus inhibiting the fungal growth and lack siderophore capability	➢ Success in case report and deferasirox oral dosage is simple and convenient	➢ Limited clinical data	[62,116]
**Surgical Intervention**				
Rhino-orbito- cerebral infection; Soft tissue infection; and localized pulmonary lesion	A critical component of effective therapy is prompt surgical debridement, which should be repeated if required. When surgery is required and feasible, it must be robust. Because the Mucorales hyphae may spread infection swiftly, it is important to remove not just necrotic tissues but also infected healthy-looking tissues in the surrounding area.	➢ Significantly increase the survival and success rate	➢ Expensive	[117,118,119]
